# Structural and Functional Connectivity Changes Beyond Visual Cortex in a Later Phase of Visual Perceptual Learning

**DOI:** 10.1038/s41598-018-23487-z

**Published:** 2018-03-26

**Authors:** Dong-Wha Kang, Dongho Kim, Li-Hung Chang, Yong-Hwan Kim, Emi Takahashi, Matthew S. Cain, Takeo Watanabe, Yuka Sasaki

**Affiliations:** 1 Department of Neurology, Asan Medical Center, University of Ulsan College of Medicine, 88 Olympic-ro 43-gil, Songpa-gu Seoul, 05505 South Korea; 2 Department of Cognitive, Linguistic and Psychological Sciences, Brown University, 190 Thayer Street - BOX 1821, Providence, RI 02912 USA; 3 Division of Newborn Medicine, Department of Medicine, Boston Children’s Hospital, 1 Autumn st. AU 453, Boston, MA 02215 USA; 4 Present Address: U.S. Army Natick Soldier Research, Development, and Engineering Center, Natick, MA 01760 USA; 5 Present Address: Education Center for Humanities and Social Sciences and Institute of Neuroscience, National Yang-Ming University, No. 155, Sec. 2, Linong St, Taipei City, 112 Taiwan

## Abstract

The neural mechanisms of visual perceptual learning (VPL) remain unclear. Previously we found that activation in the primary visual cortex (V1) increased in the early encoding phase of training, but returned to baseline levels in the later retention phase. To examine neural changes during the retention phase, we measured structural and functional connectivity changes using MRI. After weeks of training on a texture discrimination task, the fractional anisotropy of the inferior longitudinal fasciculus, a major tract connecting visual and anterior areas, was increased, as well as the functional connectivity between V1 and anterior regions mediated by the ILF. These changes were strongly correlated with behavioral performance improvements. These results suggest a two-phase model of VPL in which localized functional changes in V1 in the encoding phase of training are followed by changes in both structural and functional connectivity in ventral visual processing, perhaps leading to the long-term stabilization of VPL.

## Introduction

Visual perceptual learning (VPL) is defined as long-term visual performance improvements after visual experiences, and is thought to reflect brain plasticity in adults^1–3^. However, the underlying neural mechanisms of VPL are not completely understood^4^. The brain area(s) that change in association with VPL remain hotly debated in the field. Some researchers have suggested that VPL takes place in the early visual cortex, citing that VPL is specific to the trained feature/location. For example, VPL of a texture discrimination task is specific to the trained visual field quadrant and does not transfer to other quadrants^5^. Such location specificity suggests the involvement of the early visual cortex, in which visual processing occurs locally^5–7^. A number of human functional magnetic resonance imaging (fMRI) studies have documented the involvement of the primary visual cortex (V1)^8–11^ in VPL. On the other hand, the majority of animal studies have failed to find evidence of the involvement of V1 neurons, in contrast to results from human studies^12–14^ (but see refs^15–18^).

The results of a recent study suggest a reason for the discrepancy^7^, namely, a difference in time course of learning development. Yotsumoto *et al*.^7^ showed that in the early phase of VPL training, performance increases are accompanied by activation enhancement in the region of V1 corresponding to the trained stimulus. However, in the later phase of VPL training, after performance increases have reached a plateau, V1 activation returns to baseline levels, even while performance on the trained task remains high. These results are in accord with a two-phase model of VPL, in which V1 is involved in the encoding of VPL, and in a later phase different regions and/or aspects of the brain are involved in the long-term retention of VPL. This model accounts for both the involvement of V1 in human studies and the absence of V1 activation changes in monkey single-unit recording studies, and may effectively resolve the discrepancy between the brain areas involved in VPL. Monkey studies typically employ a much longer training period (months) as compared to human studies (days). Thus, it is possible that human studies reflect brain activity in the early phase of VPL, while monkey studies measure brain states during the later stage of VPL, in which performance increases are saturated.

Performance gains from VPL can be retained for months or years in humans^19,20^. This raises an important question: How are high levels of performance retained after performance saturation? In our previous study,^7^ no areas other than V1 showed significant dynamic blood-oxygenation level dependent (BOLD) changes over the time course of training. Thus, we hypothesize that between the early and later phases of VPL training, plasticity mechanisms that can be measured by BOLD signal changes in V1 transition to other mechanisms in which neural correlates of VPL are not visible as changes in BOLD signal amplitudes.

Thus, in the present study, we tested whether connectivity between V1 and more anterior regions changes in the later phase of VPL training. Especially, as for the connectivity between V1 and other regions, we examined white matter connectivity using diffusion tensor imaging (DTI) and functional connectivity by BOLD signals.

## Results

### Performance change

**Figure 1:**
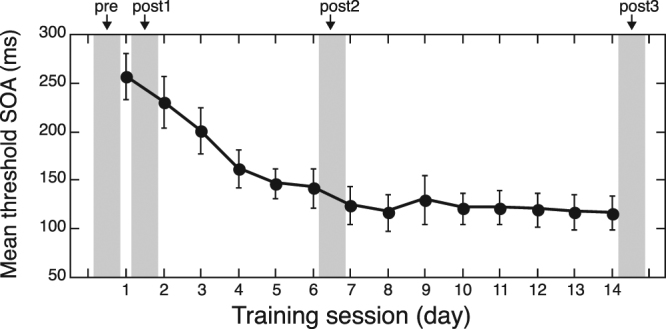
Performance improvements over training (mean ± S.E.M, *n* = 7). Shadowed areas indicate the times when the MRI scans were taken (pre-, post1-, post2- and post3-training, respectively).

Subjects were trained on a texture discrimination task (TDT), a standard VPL task for 14 sessions over the course of 3–4 weeks. Training on TDT was confined within the same visual field quadrant (either the upper left or upper right) for each subject. There were four MRI sessions: pre-training, mid-training (twice) and post-training (hereafter referred to as pre-, post1-, post2-, and post3-training, see Methods below), as shown in Fig. 1. Performance of the task gradually improved during the early training sessions and reached a plateau in later sessions (Fig. 1), consistent with previous research^7^. On average, performance reached a plateau on the 6th or 7th day of the training sessions, which roughly aligned with the post2-training MRI session.

### Track counts of white matter tracts

**Figure 2:**
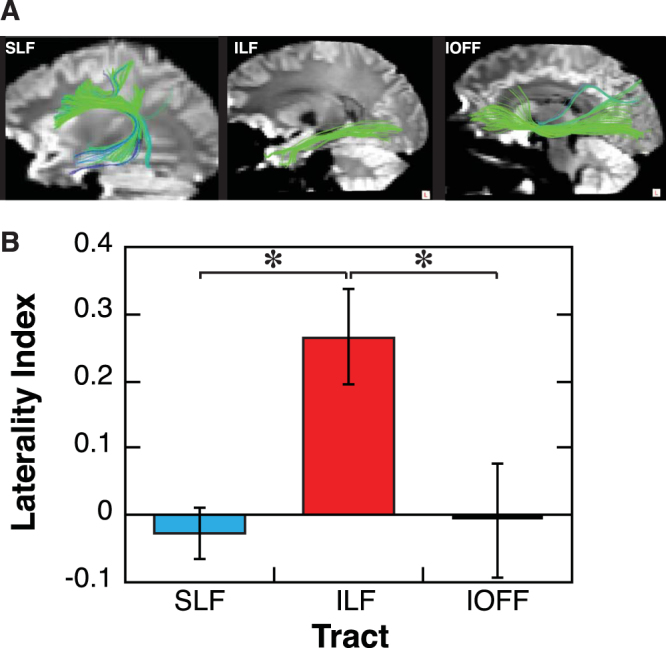
(**A**) The three major white matter tracts (green/blue) identified in the present study. (**B**) Laterality Index for each tract. Mean ± S.E.M (*n* = 7). *p < 0.05. The laterality index was computed as (L−R)/(L + R), where L and R denote the track counts for the left and right hemispheres, respectively.

We examined white matter tracts, which are important subcortical structures that connect spatially remote areas of the brain and enable rapid and efficient transfer of information. To measure possible changes in white matter structure, we used diffusion tensor imaging (DTI), which is an MRI technique that measures the random motion of water molecules in biological tissues^21^. Tractography^22–25^ applied to DTI data allows us to reconstruct major cerebral white matter pathways in the living human brain non-invasively by successively following the path of the preferred direction of water diffusion. DTI tractography was used to identify three major white matter tracts: the inferior longitudinal fasciculus (ILF), the superior longitudinal fasciculus (SLF), and the interior occipito-frontal fasciculus (IOFF) (Fig. 2A). These were reliably traced by a deterministic tractography algorithm^26^. These tracts were targeted because they are known to connect the early visual areas and the anterior parts of the brain, and to be involved in visuospatial processing^26^.

First, we measured track counts of the tracts during the pre-training MRI session. The track count represents the number of streamlines composing a particular tract, and can be compared quantitatively between subjects. The track count between distant regions-of-interest (ROIs) reflects how coherently thick the tract is. Intriguingly, the visual pathways may be asymmetrical across the hemispheres (anatomically and/or functionality), and this asymmetry is more prominent in more anterior areas than in the early visual cortex^27–29^. For example, the impact of transcranial magnetic stimulation (TMS) is stronger in the left hemisphere^28,29^. Therefore, during the pre-training MRI session, we tested whether track counts showed evidence of laterality (i.e., asymmetry) between the hemispheres. A 2-way repeated measures ANOVA (factors = Tract and Hemisphere) revealed a significant main effect of Tract (*F*(2, 12) = 13.344, *p* = 0.0009), and a borderline significant interaction between Tract and Hemisphere (*F*(2, 12) = 3.753, *p* = 0.0542), but no significant main effect of Hemisphere (*F*(1, 12) = 0.685, NS). As is shown in Fig. S1, the meaningful interaction comes from the laterality of the ILF. We then computed the lateralized index, where positive values indicate left-side dominance and negative values indicate right-side dominance (Fig. 2B). A one-way ANOVA (factor = Tract) revealed a significant main effect of Tract on laterality (*F*(2, 12) = 5.249, *p* = 0.023). Further post-hoc *t*-tests using Ryan’s multiple comparison method showed a significant difference in laterality between ILF and SLF (*t*(12) = 2.899, *p* = 0.0133), and between the ILF and the IOFF (*t*(12) = 2.703, *p* = 0.0192), but not between the SLF and the IOFF (*t*(12) = 0.196, *p* = 0.8479). These results suggest that the ILF is significantly lateralized and that the ILF is more dominant in the left hemisphere for most of the subjects.

To test whether this dominance had any behavioral relevance, we computed the correlation coefficient between performance levels on the first day of training (as quantified by the stimulus-to-mask onset asynchrony corresponding to 80% performance accuracy, see behavioral training in Methods) and the track count of the dominant ILF (i.e., the ILF in the hemisphere with the larger track count). There was a strong correlation between the track count of the dominant ILF and initial performance (*r* = −0.75, *p* = 0.0526, Fig. S2). In contrast, neither the dominant SLF nor IOFF showed significant correlations (SLF, *r* = −0.10; IOFF, *r* = −0.19). The results suggest that the dominant ILF is relevant to performance on this type of visual task.

### Fractional anisotropy change

Next, we measured fractional anisotropy (FA) from the identified tracts in each hemisphere (see Methods, below). FA quantifies the directionality of water diffusion as a normalized value between zero and one. A value of zero means that diffusion is isotropic, (occurring equally in all directions), while a value of one means that diffusion occurs along only one axis. FA is therefore thought to reflect several key characteristics of white matter, including fiber density, axonal diameter, and myelination^30–32^. It has been suggested that inter-individual variations in white matter structure measured from DTI and tractography are sensitive enough to reveal inter-individual differences in behavior^30,31^.

**Figure 3:**
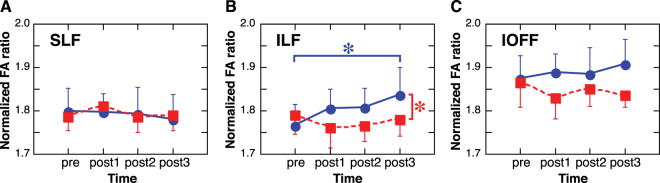
The FA changes (mean ± S.E.M., *n* = 7) of dominant (blue line) and non-dominant (red line) tracts over training. Only the ILF (**B**) showed a significant change during training. In particular, the FA of the dominant ILF significantly increased at post3 in comparison to the pre-training, and to the non-dominant ILF. *p < 0.05.

We investigated whether the FA values of the three tracks changed in a manner consistent with long-term VPL. Since the dominant ILF showed strong behavioral relevance even before the start of training, we analyzed the FA of the ILF across sessions for both the dominant and non-dominant sides. A 2-way ANOVA with repeated measures (factors = Dominance (dominant or non-dominant) and Time (pre, post1, post2, and post3-training)) was conducted to see whether the FA of the ILF changed significantly during training. The FA of the ILF showed a significant interaction of factors (*F*(3, 18) = 4.885, *p* = 0.0117), while no significant main effects were found. Further analyses showed a significant main effect of Dominance at post3-training (*F*(1, 24) = 4.627, *p* = 0.0418), and a significant main effect of Time in the dominant tract (*F*(3, 36) = 3.023, *p* = 0.0421, Fig. 3) driven by an increase in FA between the pre- and post3-training (*t*(36) = 2.996, *p* = 0.0049, Ryan’s multiple comparison correction method; Fig. 3). The results show that the FA of the dominant ILF significantly increased, with significant differences in FA between the dominant and non-dominant ILF at post3-training. In contrast, 2-way repeated measures ANOVAs did not reveal any significant main effects of Dominance, Time, or interactions between them in either the SLF or the IOFF.

The increase in FA over training in the dominant ILF was not due to the laterality of the visual field used for training. We sorted the brain hemispheres based on whether they represented the trained or untrained visual field. For example, if the stimulus was presented in the left visual field, then the tracts in the right hemisphere were classified as the trained side, and the left hemisphere the untrained side. A two-way repeated-measures ANOVA (factors = Side (Trained/Untrained), and Time) did not reveal any significant FA changes (Fig. S3). Together, these suggest that the significant FA change that occurred in the dominant ILF was independent of the location of the trained visual field.

### Functional connectivity changes

Although it is crucial to clarify how structural changes are related to functional changes, most studies have shown only one of them without investigating the relationship between the two types of changes. Since the FA of the dominant ILF demonstrated significant changes suggesting structural changes over the course of VPL, we wished to investigate how these changes might correspond to changes in functional connectivity^33–37^ between V1 and the cortical regions that are connected by the tract in later phases of VPL. Functional connectivity is measured as the correlation between temporal fluctuations in the BOLD signals of two brain regions^33–37^. There is some precedent for such change; it has been found that functional connectivity between V1 and the frontal eye field is changed in association with VPL of a letter identification task^38^.

The ILF runs between the occipital lobe and the lateral/medial temporal cortex^26^. Therefore, we studied the functional connectivity between V1 and the anterior cortical areas, which are connected by the dominant ILF, using V1 as the seed region. Seven targeted regions were selected as destination areas of the ILF using Brodmann areas (BA): the temporopolar area (BA38), inferior temporal gyrus (BA20), middle temporal gyrus (BA21), insula (BA13), parahippocampal gyrus (BA36), anterior entorhinal cortex (BA34), and posterior entorhinal cortex (BA28).

**Figure 4:**
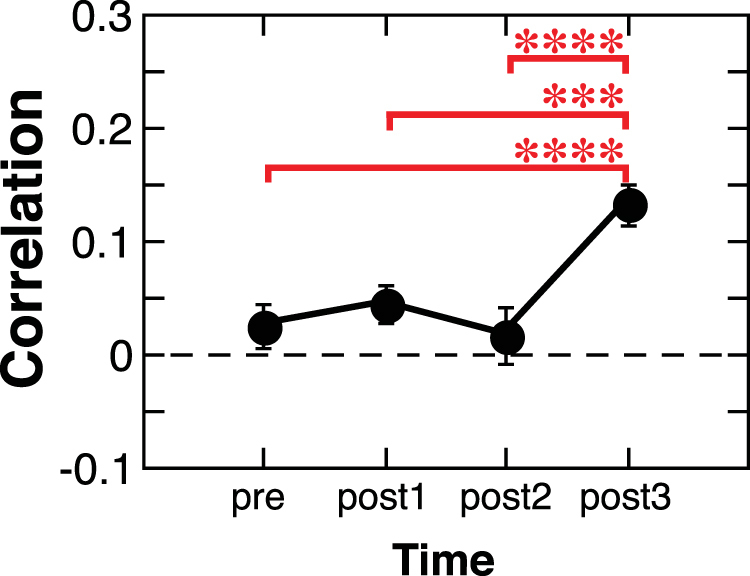
The functional connectivity averaged across 7 pathways along the dominant ILF between V1 and the lateral/medial temporal areas over the course of perceptual learning training (mean ± S.E.M., *n* = 7). The y-axis shows the correlation coefficients transformed into z-scores. ***p < 0.005, ****p < 0.001. See Fig. S4 for each pathway.

We calculated the functional connectivity (i.e., correlation coefficients) for seven pathways between V1 (BA 17) and the anterior regions along the ILF listed above at pre-, post1-, post2- and post3-training. Next, we investigated whether the functional connectivity was significantly changed over the course of training by applying two-way repeated measures ANOVA (factors = Pathway and Time). The correlation coefficients that represent functional connectivity were z-transformed to account for variance instability inherent to measures of correlation. The results showed significant main effects of Pathway (*F*(6, 36) = 7.291, *p* < 0.001), and Time (F(3, 18) = 9.338, p = 0.0006), without any interaction (*F*(18, 108) = 1.294, NS). Post hoc tests (multiple-comparison corrected with Ryan’s method) to investigate which pairs of time points were significantly different (Fig. 4) revealed significant differences in the mean functional connectivity between the pre- and post3-training (*t*(18) = 4.349, *p* = 0.0003), post1- and post3-training (*t*(18) = 3.534, *p* = 0.0023), and post2- and post3-training (*t*(18) = 4.731, *p* = 0.0001). The results indicate that the functional connectivity between V1 and the anterior regions along the dominant ILF was significantly increased at post3-training. See Fig. S4 for functional connectivity for each of the seven pathways.

Is the increased functional connectivity between the V1 and the anterior regions associated with increased BOLD activation in these anterior regions? To address this question, we tested whether the BOLD signal, that was obtained by subtraction between the task period and rest periods, in each of these 7 destination ROIs was greater at post-3 training session than at the pre-training session (Fig. S8). We found that the BOLD signal in none of the 7 destination ROIs was significantly different between post-3 and pre-training sessions (all p > 0.227). These results suggest that the increased functional connectivity as well as increased performance in the task in the later phase of training was not due to increased BOLD activation in these regions.

Does the functional connectivity between V1 and the anterior regions along the dominant ILF at post3-training have performance relevance? We computed the behavioral performance improvement between pre- and post3-training. Next, we computed the mean of the z-transformed correlation coefficients between V1 and the seven pathways along the dominant ILF. The correlation between the performance improvement and the mean functional connectivity between V1 and the anterior regions along the dominant ILF at post3-training was significant (*r* = 0.75, *p* = 0.049, Fig. S5B). Thus, the result indicates that the functional connectivity between V1 and the anterior regions along the dominant ILF has a strong behavioral relevance.

Is the functional connectivity between V1 and the anterior regions along the dominant ILF associated with changes in any white matter properties? Interestingly, the track count of the dominant ILF computed at pre-training was strongly and significantly correlated with the mean functional connectivity between V1 and the anterior regions along the dominant ILF at post3-training (*r* = 0.82, *p* = 0.023, Fig. S5A). This result suggests that certain white matter properties may predict functional connectivity changes along the dominant ILF in the later phase of VPL, which is correlated with performance improvement. Thus, the white matter structure of the dominant ILF may mediate changes in functional connectivity and long-term visual plasticity.

## Discussion

The present study clearly supports our hypothesis that the structural and functional connectivity between V1 and anterior parts of the brain change along the dominant ILF in the later phase of VPL, after behavioral performance reaches a plateau. Previously, we showed that V1 activation is enhanced in early phases of VPL, when behavioral performance is quantifiably improving^7^. However, V1 activation returns to baseline levels in later phases of training, when behavioral performance reaches its plateau and remains high^7^. Taken together with these findings, the present study suggests a two-phase model of VPL: in the earlier phase of training, encoding of VPL involves retinotopically localized regions of V1 and ends when behavioral performance is saturated, while in the later phase, retention of VPL is subserved by the enhanced structural and functional neural connectivity between the visual cortex and the lateral/medial temporal cortex.

What do these neural changes in the later phase of training represent? Previously, we suggested that the enhanced BOLD activation seen in the trained region of V1 in the early phase of VPL training is associated with the synaptic re-organization and optimization^7^, which leads to the trained location specificity of learning. The increased connectivity along the dominant ILF in the later phase of VPL training observed in the present study may represent the enhancement of mid-level complex visual processing in the ventral visual pathway^39^, including the judgment of the orientation of the textured objects—which is an essential process in the present task—or the reweighting process of the network between sensory areas and decision areas, as suggested by other research^2,40^. We propose that such mid-level visual processing or reweighting process may be involved in the long-term retention of VPL^19,20^.

Importantly, as mentioned above, BOLD activation in the later phase of VPL training was not significantly increased in the anterior temporal regions, which are destination areas in the pathways. This suggests that the increased connectivity between the V1 and the anterior temporal regions in the later phase is not directly associated with increased BOLD activation in the anterior temporal regions. Functional connectivity changes in a pathway are indexed as changes in the correlation between the areas connecting the pathway and theoretically can occur without changes in the averaged BOLD signal from the area in either end of the pathway.

Karni and Sagi have shown that performance improvements on a texture discrimination task demonstrate complete interocular transfer when measured within the training block, but that the degree of transfer is greatly reduced if measured several hours after training^5,19^. These results suggest that VPL initially takes place in a binocular stage and moves to a lower monocular stage as learning proceeds. Such a hypothesis does not contradict the present finding nor our two-phase model of VPL, because the timescales of the two studies are significantly different: the locus-shift observed in the Karni and Sagi study occurs on the order of hours, whereas the putative locus-shift of the present study occurs on the order of weeks. The activation enhancement in the local region of V1 in the Yotsumoto study (2008) may reflect the involvement of the lower monocular stage a few hours after training, thus corresponding to the Karni and Sagi studies. It seems that the locus of VPL may shift over several regions during the complete time course of its development.

Important changes in structural and functional connectivity that manifested in a later phase of VPL took place along the dominant ILF. Previous research has suggested that the ILF is involved in mid-level visual functions and reading skills^26,27,31,41–44^. Ortibus *et al*. investigated whether the integrity of the ILF was associated with deficits in visual perception and object recognition, especially in children^27^. They found that a decrease in FA values in the ILF was significantly correlated with impairment in visual tasks compared to typically developing children. Moreover, the children with impairments in visual perception showed consistently lower FA values in the left hemisphere. Thus, they suggest that the left ILF may be an essential part of normal ventral-stream visual processing^27^. Interestingly, a significant lateralization was also found in the ILF in the present study with healthy young adults. Properties of the dominant ILF, which was usually in the left hemisphere, were associated not only with the initial level of performance of the task, but also with the degree of performance improvement in the prolonged training period. Taken together, these results suggest that the integrity of ILF on the dominant side plays a critical role in developmental visual impairments and visual plasticity in young adults.

However, the reason for the lateralization of the ILF is not clear. We do not think that the lateralization of the ILF was related to handedness, or the trained visual field quadrant. In our data, handedness may not be correlated with lateralization of ILF. There was only one subject who was a left-hander. As shown in Fig. S6, the ILF lateralization in the left-hander does not seem to be deviated from other right-handers. In addition, the side of the trained visual field quadrant is not likely to be related to the ILF lateralization, given that the trained visual field quadrant was counterbalanced across subjects, while the dominant ILF tends to be the left side. Future systematic investigations need to be conducted to clarify the reason for the lateralization of the ILF.

One may wonder why the lateralization of SLF is absent in the study, given that the arcuate fasciulus (AF), which is a part of SLF, plays a critical role in language processing^27,31,43,44^, for which many studies have reported predominantly left lateralized structures^45^. The answer may be found in our procedure to identify SLF. There are several ways to classify the SLF and AF. One way is to sub-divide the SLF into three parts^46^, SLF I, SLF II and SLF III, which corresponds to AF. SLF I/II is involved in the perception of visual space, whereas SLF III is involved in language processing^46^. Our identification^26^ of SLF included all the three SLF subdivisions, and this may be why we did not see left lateralization in SLF.

The present study shows that the ILF lateralization plays a key role in the long-term VPL development. However, this does not dismiss involvements of other structures that are not investigated in the present study. For instance, the optic radiation that connects the LGN and V1 may play a critical role in the long-term VPL development^47^. However, we could not reliably measure the optic radiation, since it is curved too steeply for the current tracking system used in the study to track precisely.

In the present study, we examined how structural and functional connectivity between V1 and higher areas are changed in association with the long-term retention of behavioral performance enhancements on a visual perceptual learning task. To our knowledge, this is the first brain imaging study that indicates coordinated functional and structural changes in association with VPL. Given that many types of VPL have common characteristics including high specificity in the trained features and location and long retention, it is possible that similar functional and structural changes at later phases of training occurs in other types of VPL. We need to wait for a future research to examine this interesting possibility.

## Methods

### Subjects

A total of 7 young subjects (4 females and 3 males, 20–29 years old, mean = 25.9 ± 2.79 SD) participated in the study. All subjects had normal or corrected-to-normal vision and gave written informed consent for their participation in the experimental protocol approved by the Institutional Review Board of Brown University. All experiments were performed in accordance with relevant guidelines and regulations.

### Behavioral training

The texture discrimination task (TDT)^5–7,48^ was employed for the behavioral training. On each trial, visual stimuli were presented on a CRT screen at a viewing distance of 57 cm. A test stimulus was presented briefly (13 ms), followed by a variable-duration blank screen and then a mask stimulus (100 ms). The stimulus-to-mask onset asynchrony (SOA) between the target display and the mask display was varied across blocks. The test stimulus consisted of a centrally located letter, either “L” or “T,” and a peripherally positioned horizontal or vertical array of three diagonal lines—the target array—on a background of horizontal lines. While keeping their eyes fixated on the center of the stimulus display, subjects were asked to respond twice for each trial: once to identify the letter (“L” or “T,”) and once to indicate the orientation (horizontal or vertical) of the target array by pressing two of four buttons on a response button box in order. The purpose of the letter task was to ensure subjects’ eye fixation at the center of display. During the training sessions, the horizontal or vertical target array was presented only in the designated visual field quadrant (i.e., the trained visual field). The trained visual field was counterbalanced across subjects; either in the upper right quadrant only (*n* = 3) or the upper left quadrant only (*n* = 4). Each training session contained 840 trials presented in 7 blocks. Each block contained 120 trials with a constant SOA. Each training session started with the 500 ms SOA and decremented it on each subsequent block: 250, 200, 150, 100, 67, and 50 ms. Logistic functions were fitted to each subject’s resulting psychometric curve, and the SOA value corresponding to 80% performance accuracy was taken as a threshold measure for each session.

### MRI sessions

There were 4 separate MRI sessions: before the onset of training, and after the 1st, 6th and 14th training sessions (pre-, post1-, post2- and post3-training, respectively). During the MRI sessions, we collected diffusion tensor imaging, anatomical imaging, and functional imaging data.

### Diffusion tensor imaging (DTI)

Diffusion-weighted and non-weighted MR images were collected from all subjects using a single-shot, twice-refocused spin echo sequence with automatic alignment of the slices parallel to the intercommissural plane. Parameters for diffusion MRI acquisition were as follows: TR = 8.0 s; TE = 84 ms; matrix size = 128 × 128; field-of-view = 256 × 256 mm; slice thickness = 2 mm; interslice gap = 0 mm; number of acquisitions = 70 (60 non-collinear directions with *b-*value 700 s/mm^2^ and 10 with *b-*value 0 s/mm^2^); voxel size = 2 × 2 × 2 mm^3^; and number of slices = 64. These images were used for DTI tractography. The diffusion-weighted and non-weighted MR images volumes were corrected for motion and eddy current distortion using FMRIB’s Linear Image Registration Tool (FLIRT)^49^.

For fiber tracking, a deterministic algorithm was chosen because this study pre-specified ROIs as anatomically well-established white matter tracts involved in visuospatial processing. Moreover, deterministic fiber tracking measurements are known to provide a reliable tool for quantitative longitudinal evaluations of diffusion properties in major, thick white matter pathways^50^. Diffusion Toolkit and TrackVis software (www.trackvis.org) were used for reconstruction, fiber tracking, visualization, and analysis of diffusion data as follows. First, visual inspection of the diffusion-weighted images acquired from each subject revealed stable head position without artifacts. Second, diffusion tensor maps including FA maps were reconstructed from the diffusion image volumes using a reconstruction program implemented in Diffusion Toolkit. Third, whole-brain fiber tracks were generated from the diffusion tensor maps using a fiber assignment by continuous tracking (FACT) algorithm^22–25^ implemented in Diffusion Toolkit. Tracking followed the direction of preferred water diffusion (the direction of eigenvector with the maximum eigenvalue) in 0.5 mm step lengths, and was terminated if the angle between two consecutive directions of preferred water diffusion, or consecutive eigenvectors with maximum eigenvalues, was greater than 45°, or if the FA value was less than 0.15. We used the threshold of FA < 0.15, because it has been reported to provide the best tradeoff between fewer erroneous tracts and penetration into the white matter^51^.

The visualization program (TrackVis) allowed us to apply multiple track filters to select, display, and analyze specific fiber bundles. We identified three long white matter tracts (Fig. 2A): superior longitudinal fasciculus (SLF), inferior longitudinal fasciculus (ILF), and inferior occipito-frontal fasciculus (IOFF). The SLF includes three segments (SLF I, SLF II, and SLF III), which is a dorso-ventral associative bundle connecting the perisylvian cortex of the frontal, parietal and temporal lobes. The ILF is a ventral associative bundle connecting visual areas to the lateral/medial temporal lobe. The IOFF is a ventral associative bundle that connects the ventral occipital lobe and the ventral frontal lobe including the orbitofrontal cortex^26^.

All fibers were identified using a ROI approach^26^. ROI delineation and fiber tracking were done blinded to the subjects’ information (e.g., trained side, performance level, etc.) by an expert neurologist (D.-W.K) who has extensive neuroanatomical knowledge. In brief, a single ROI approach on the dorsal part of the fasciculus was used for the dissection of SLF. A two-ROI approach was used to dissect the fibers of ILF and IOFF. The first ROI was defined around the white matter of the occipital lobe for both ILF and IOFF. The other ROI was defined around the white matter of the anterior temporal lobe for ILF, and around the external/extreme capsule for IOFF. Additional ROIs were used to remove fibers that did not belong to the tracts investigated in this study. It was important to distinguish between the fibers of ILF and those of IOFF.

In addition, great care was taken to standardize the placement and size of the ROIs bilaterally in each subject. Because we had diffusion scans at 4 time points, we had to avoid variations in manual ROI placement and make sure that the size, shape and location of ROIs were consistent across all time points within each subject. Because alignment of diffusion images to the standardized space may result in loss of information on individuality, we spatially registered ROIs instead of transforming DTI data. In other words, we registered ROIs of the first diffusion scan to the second, third and fourth diffusion scans in each subject, and then performed tractography on the native space at each time point. For the ROI registration, FLIRT^49^ was again used.

FA value and the number of tracks in each tract were obtained using TrackVis. We normalized the mean FA value of each tract to that of the whole brain (i.e., calculated as the mean FA of the tract divided by the mean FA of the whole brain) to reduce potential sources of variability in diffusion MRI measures, including changes in the MRI scanner environment from scan to scan. Because of right-left asymmetry in the number of tracks, we determined dominant side as the side with a larger number of tracks in each fasciculus.

### MRI-Anatomical scan

For the anatomical reconstruction^52^, three T1-weighted MR images (MPRAGE) were acquired (TR = 2.531 sec, TE = 3.28 ms, flip angle = 70°, TI = 1.1 sec, 256 slices, voxel size = 1.3 × 1.3 × 1.0 mm^3^, resliced during analysis to 1 mm^3^).

### Functional connectivity

Functional connectivity was calculated using BOLD signals during the rest periods that were inserted during the TDT task. That is, we analyzed the BOLD signals when only the fixation point was presented against the dark background. In contrast to the rest period results, when we computed the functional connectivity using BOLD signals during the task periods, no significant change was found over time (see Fig. S7 for more detail).

The TDT design used during the MRI session was slightly modified from the one used during the behavioral training. One fMRI session consisted of 8 runs with 128 trials per run. We presented 128 trials for each of the two visual locations using an event-related fMRI paradigm. Half of the 128 trials were fixation trials. After a 500 ms fixation cross, a target array was presented for 20 ms followed by a 100 ms mask. Subjects were asked to respond to a fixation letter and an orientation of the target array by pressing buttons in order on a box that they held in their hands. Immediate auditory feedback was given only for the fixation letter task. A constant SOA of 100 ms was used during the task in the scanner. The location of target presentation was in either the upper left or upper right visual field quadrant. One of the visual field quadrants corresponded to the one used during the behavioral training sessions.

Functional images were acquired on a 3 T Siemens Trio whole-body scanner using a 12-channel head coil. Functional images were collected with a gradient echo EPI sequence (TR = 2.0 s, TE = 30 ms, flip angle = 90°). Thirty-five contiguous slices (3 × 3 × 3.5 mm^3^) oriented parallel to the AC-PC plane were acquired to cover the entire brain.

Correlation analysis was performed using the Functional Connectivity toolbox (CONN; http://web.nitrc.org/projects/conn/) to compute functional connectivity. Functional MRI data were first preprocessed in SPM8 using slice time correction, realignment, coregistration to the structural image, spatial normalization to the Montreal Neurological Institute (MNI) template, and spatial smoothing with a Gaussian kernel of 8 mm full width at half maximum. The linear detrend was performed during preprocessing. After pre-processing, images were high-pass filtered with cutoff frequency of 0.01 Hz and motion regressed to reduce the influence of noise. Since we were interested in the functional connectivity changes between the primary visual cortex (BA17) and other cortical regions that are connected by the ILF, seven regions-of-interest (ROIs) were selected based upon Brodmann areas (BAs). The seven ROIs were BA13, BA20, BA21, BA28, BA34, BA36, and BA38, located at the medial and lateral temporal regions. We calculated a temporal correlation between the BOLD signals from the seed region (BA17) to all other ROIs, by computing the average BOLD time series across all the voxels within each ROI. White matter, cerebrospinal fluid (CSF), and physiological noise source reduction were taken as confounds, following the implemented CompCor strategy^53^. Correlation coefficients were transformed into Fisher Z-scores for the subsequent ANOVA. False discovery rate (FDR) correction^54,55^ was applied to the statistical values of ANOVA to correct for multiple comparisons for further analyses (Fig. S4).

## Electronic supplementary material

Supplementary information
