# Supplementary material for: Structural and Functional Connectivity Changes Beyond Visual Cortex in a Later Phase of Visual Perceptual Learning
Source: Sci Rep. 2018 Mar 26;8:5186. doi: 10.1038/s41598-018-23487-z (PMC5979999; doi:10.1038/s41598-018-23487-z)
Supplement: Supplementary file 1 — Supplementary information [file 41598_2018_23487_MOESM1_ESM.pdf]

Supporting Information

Structural and Functional Connectivity Changes Beyond Visual Cortex in a Later Phase of Visual Perceptual Learning

AUTHORS
Dong-Wha Kang, Dongho Kim, Li-Hung Chang, Yong-Hwan Kim, Emi Takahashi, Matthew S. Cain, Takeo Watanabe, Yuka Sasaki

FIGURE S1

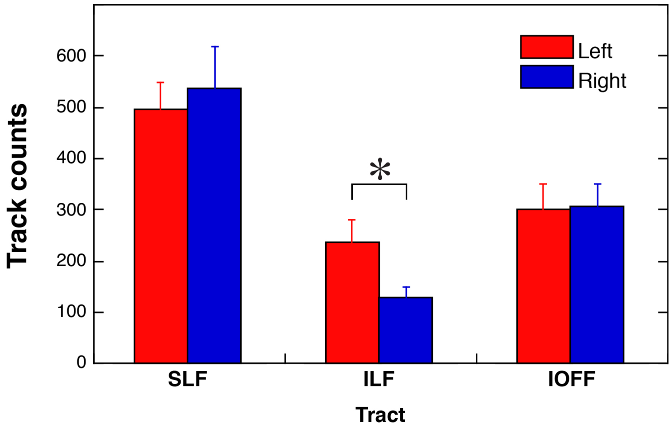

Fig. S1. The track counts for each tract in each hemisphere. The ILF shows the significant difference between the left and right hemispheres. Paired t-test revealed significant laterality in the ILF ($t(6)=2.8479$, $p=0.029$), but not in SLF or IOFF.

FIGURE S2

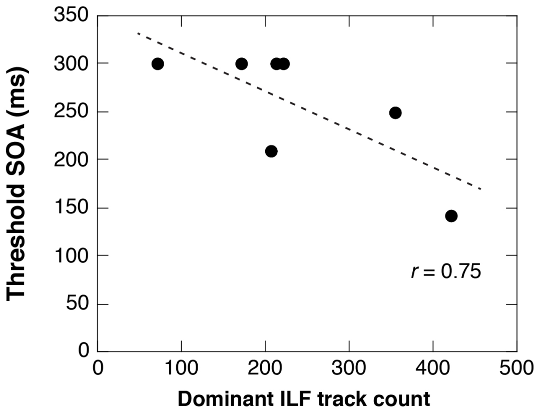

Fig. S2. The correlation between the initial 80% threshold SOA at the first day of behavioral training and the track count of the dominant ILF (n=7).

FIGURE S3

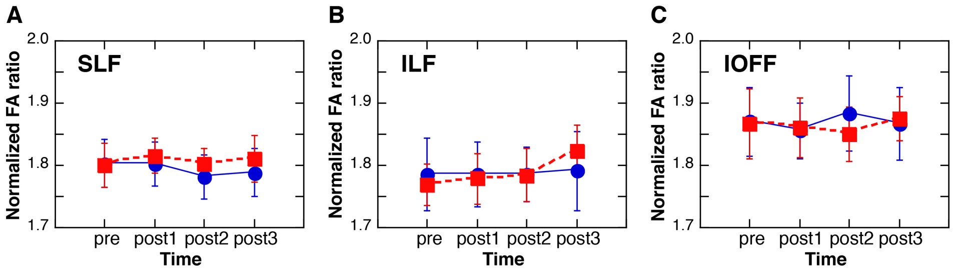

Fig. S3. Non significant FA changes (mean ± S.E.M) of the trained side (blue) and the untrained side (red) of tracts over training in the SLF (A), ILF (B), and IOFF (C).

FIGURE S4

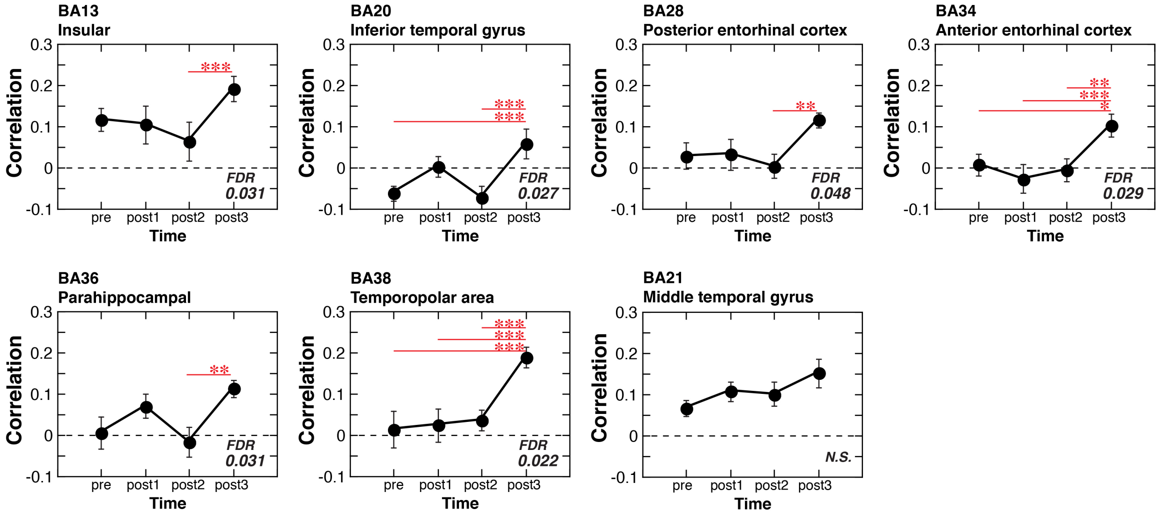

Fig. S4. Functional connectivity in seven pathways of the dominant ILF over time. For each panel, the x-axis shows the session and the y-axis the correlation coefficients transformed into z-scores (mean ± S.E.M., n = 7). Each of the seven pathways was separately analyzed with a one-way repeated measures ANOVA (factor = Time), after a two-way repeated measures ANOVA (factors = Pathway and Time) revealed significant main effects of both Pathway and Time on the functional connectivity along the dominant ILF (see the text and **Fig. 5** in the main text). Six out of seven pathways showed a significant main effect of Time, after controlling for false discovery rate (FDR)[1, 2]. Red asterisks indicate significantly different pairs of time points by post- hoc tests using Ryan's multiple correction method on each of the six pathways where the Time effect was significant. In each of these six pathways, the functional connectivity increased at post3-training. **p<0.01, ***p<0.005.

FIGURE S5

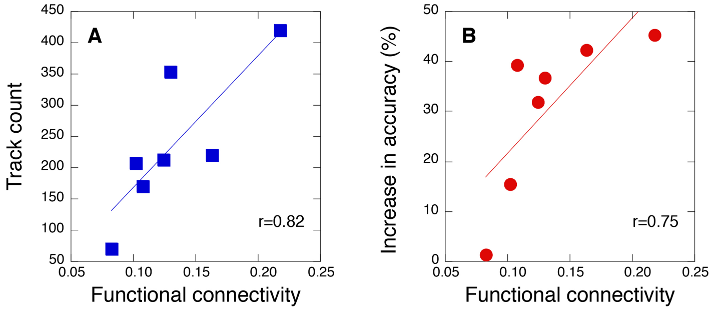

Fig. S5. Correlations among functional connectivity, track count, and performance improvement. The x- axis shows the functional connectivity averaged across the seven pathways along the dominant ILF at post3-training. (A) Scatter plots between the functional connectivity and the track count of the dominant ILF. (B) Scatter plots between the functional connectivity and the performance improvement between the pre- and post3-training. Note that none of the data corresponds to outliers according to Grubbs' Test (alpha=0.05).

FIGURE S6

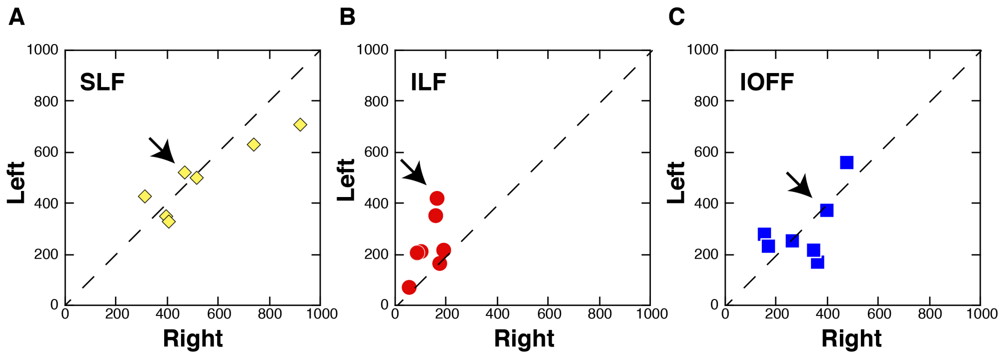

Fig. S6. Scatter plots of track counts for each tract in the left and right hemispheres individually. The x-axis shows the track counts in the right hemisphere, and the y-axis in the left hemisphere. If the track counts for the both hemispheres are identical, the data fall onto the dotted line. The track counts were significantly lateralized for the ILF. The arrow indicates the data of the left-handed subject whose data do not seem to be an outlier from others who were all right-handed.

FIGURE S7

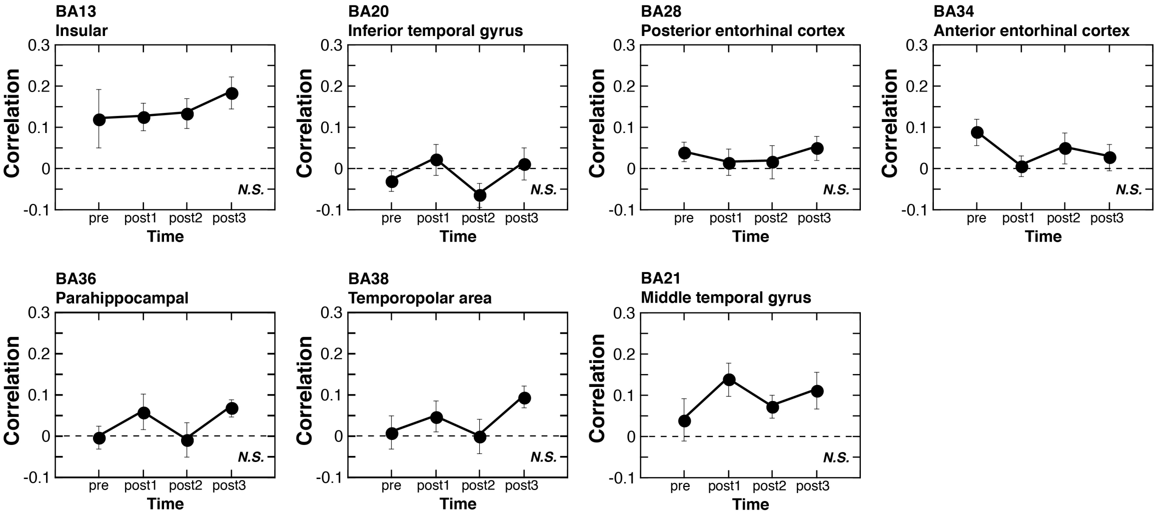

Fig. S7. Functional connectivity in seven pathways of the dominant ILF computed based on fMRI signals during the task periods showed no significant change over time. For each panel, the x-axis shows the time and the y-axis the correlation coefficients transformed into z-scores (mean ± S.E.M., n = 7). A two-way repeated measures ANOVA (factors = Pathway and Time) revealed a significant main effect of Pathway ($F(6,36)=8.518$, $p=0.0000$), but not a main effect of Time ($F(3,18)=1.252$, NS), and no interaction between them ($F(18,108)=1.182$, NS). Next, each of the seven pathways was separately analyzed with a one-way repeated measures ANOVA (factor = Time). None of them showed a significant main effect of Time. The reason we do not see significant changes in the functional connectivity while subjects were actively engaged in the task is not known. However, the actual manual responses and/or stimulus presentation might have overwhelmed the functional connectivity along the dominant ILF between V1 and the lateral/medial temporal areas, unlike in the analysis presented in Fig. S5, which was computed based on the fixation period.

FIGURE S8

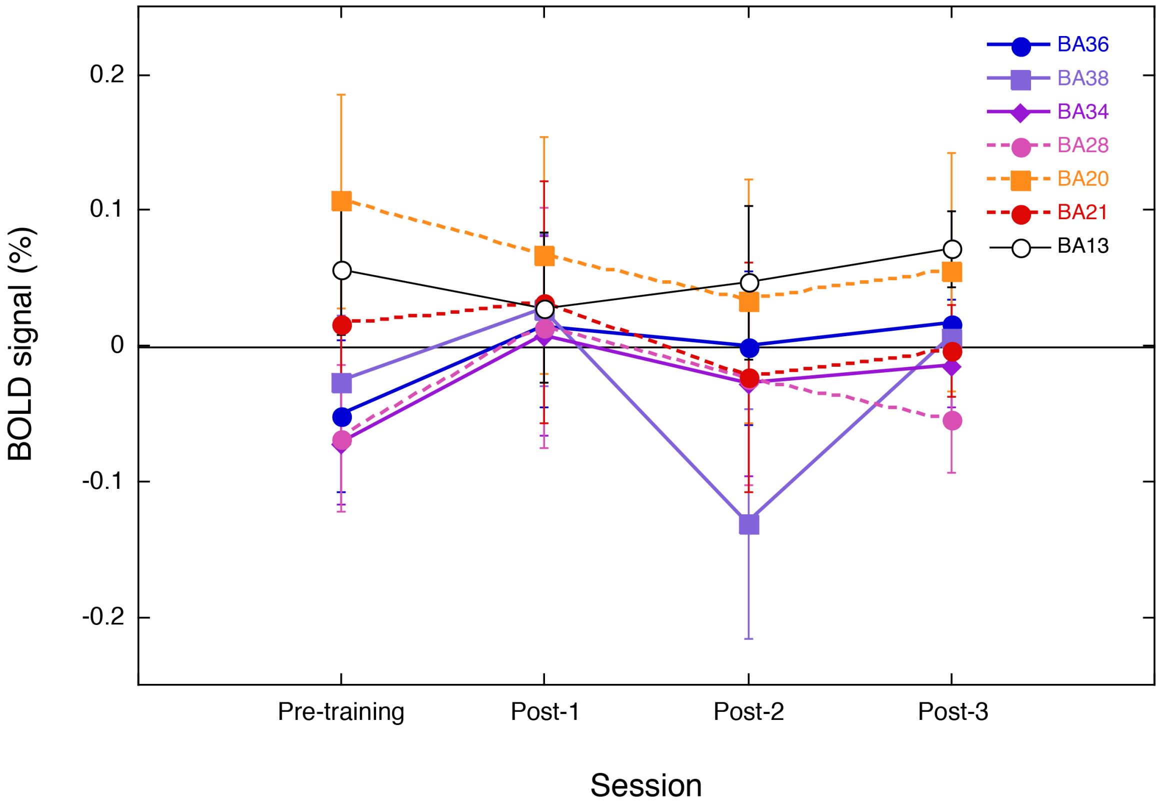

Fig. S8. The mean BOLD signals (± S.E.M) in the 7 anterior temporal ROIs. None of ROIs showed a significant difference between the pre-training and post-3 training sessions.

REFERENCES

1. Benjamini Y, Hochberg Y. Controlling the false discovery rate: a practical and powerful approach to multiple testing. *Journal of the royal statistical society Series B (Methodological)*, 289-300 (1995).

2. Genovese CR, Lazar NA, Nichols T. Thresholding of statistical maps in functional neuroimaging using the false discovery rate. *Neuroimage* **15**, 870-878 (2002).
